# Cimetidine Does Not Inhibit 5-Aminolevulinic Acid Synthase or Heme Oxygenase Activity: Implications for Treatment of Acute Intermittent Porphyria and Erythropoietic Protoporphyria

**DOI:** 10.3390/biom14010027

**Published:** 2023-12-24

**Authors:** Makiko Yasuda, Sangmi Lee, Lin Gan, Hector A. Bergonia, Robert J. Desnick, John D. Phillips

**Affiliations:** 1 Department of Genetics and Genomic Sciences, Icahn School of Medicine at Mount Sinai, 1425 Madison Ave Box 1498, New York, NY 10029, USA; sangmi.lee@mssm.edu (S.L.); lin.gan@mssm.edu (L.G.); robert.desnick@mssm.edu (R.J.D.); 2 Department of Medicine, Hematology Division, University of Utah School of Medicine, Salt Lake City, UT 84132, USA; hector.bergonia@hsc.utah.edu (H.A.B.); john.phillips@hsc.utah.edu (J.D.P.)

**Keywords:** porphyrias, acute intermittent porphyria, erythropoietic protoporphyria, disorders of heme biosynthesis, therapies for porphyrias, cimetidine, 5-aminolevulinic acid synthase, heme oxygenase

## Abstract

Acute intermittent porphyria (AIP) is characterized by acute neurovisceral attacks that are precipitated by the induction of hepatic 5-aminolevulinic acid synthase 1 (ALAS1). In erythropoietic protoporphyria (EPP), sun exposure leads to skin photosensitivity due to the overproduction of photoreactive porphyrins in bone marrow erythroid cells, where heme synthesis is primarily driven by the ALAS2 isozyme. Cimetidine has been suggested to be effective for the treatment of both AIP and EPP based on limited case reports. It has been proposed that cimetidine acts by inhibiting ALAS activity in liver and bone marrow for AIP and EPP, respectively, while it may also inhibit the hepatic activity of the heme catabolism enzyme, heme oxygenase (HO). Here, we show that cimetidine did not significantly modulate the activity or expression of endogenous ALAS or HO in wildtype mouse livers or bone marrow. Further, cimetidine did not effectively decrease hepatic ALAS activity or expression or plasma concentrations of the putative neurotoxic porphyrin precursors 5-aminolevulinic acid (ALA) and porphobilinogen (PBG), which were all markedly elevated during an induced acute attack in an AIP mouse model. These results show that cimetidine is not an efficacious treatment for acute attacks and suggest that its potential clinical benefit for EPP is not via ALAS inhibition.

## 1. Introduction

The inherited porphyrias include eight major disorders, each resulting from the defective activity of a specific heme biosynthetic enzyme [1,2]. Acute intermittent porphyria (AIP) is an autosomal dominant disorder due to the half-normal activity of hydroxymethylbilane synthase (HMBS; Supplemental Figure S1A). Symptomatic AIP patients experience life-threatening acute neurovisceral attacks that typically begin with excruciating abdominal pain and may progress to include hypertension, nausea and vomiting, peripheral neuropathy, and paralysis [1,2]. The acute attacks are precipitated by various environmental and physiologic factors that induce the hepatic expression of 5-aminolevulinic acid synthase 1 (ALAS1), the first and rate-limiting enzyme of the heme biosynthetic pathway (Supplemental Figure S1A) [3,4]. Most precipitating factors not only directly increase transcription of hepatic *ALAS1* [4,5,6] but also increase the demand for heme, either by increasing the production of cytochrome P450 (CYP) hemoenzymes (e.g., hormonal fluctuations [7,8] and CYP-inducing drugs) or by inducing the hepatic expression of the heme-degrading enzyme heme oxygenase (HO) (e.g., in fasting [9,10] and infection [11,12]). When hepatic ALAS1 is induced in AIP patients, the half-normal HMBS activity becomes rate-limiting and insufficient to meet the increased heme demand, leading to the depletion of the free heme pool. Under heme-replete conditions, the free heme pool negatively regulates *ALAS1* expression at the transcriptional [13,14] and post-transcriptional levels [15,16,17]. On the contrary, when the free heme pool is depleted, hepatic *ALAS1* is de-repressed and further induced, leading to the consequential accumulation of the porphyrin precursors 5-aminolevulinic acid (ALA) and porphobilinogen (PBG), which are thought to mediate the acute attack symptoms [18,19,20].

Currently available treatments for acute attacks decrease or prevent accumulation of ALA and PBG by suppressing hepatic *ALAS1* expression. Since its efficacy was reported in 1971, the ‘standard’ therapy for treating acute attacks has been the intravenous infusion of hemin [21,22], which is believed to act by providing exogenous heme for the negative feedback inhibition of hepatic *ALAS1*. More recently, an RNA interference (RNAi) therapeutic that silences hepatocyte *ALAS1* expression (Givosiran) has been approved in the US and other parts of the world and is currently used prophylactically in patients who suffer recurrent acute attacks [23,24,25,26].

Cimetidine is a histamine H2 receptor antagonist that is commonly prescribed for treating and preventing ulcers of the stomach and small intestine and for the treatment of gastroesophageal reflux. Previously, cimetidine was suggested to be effective for the treatment of acute porphyric attacks based on a small number of case reports [27,28,29,30]. In a total of seven AIP patients, five of whom were experiencing attacks and two of whom were in remission, cimetidine administration (800–1200 mg/day) was reported to markedly decrease urinary ALA and/or PBG and improve acute attack symptoms (among patients having an attack). However, since there have been no controlled clinical trials evaluating the therapeutic effectiveness of cimetidine, it remains unclear whether this drug truly had a positive impact on the clinical course of these patients or if clinical and biochemical improvement occurred over time, irrespective of treatment. In the context of AIP, it has been reported that cimetidine or its metabolite(s) inhibit(s) the enzymatic activity of hepatic ALAS1 and HO in rat livers [31,32]. Inhibition of the latter would increase the free heme pool and decrease transcription of the *ALAS1* gene and/or inhibit the import of the ALAS pre-protein into the mitochondria [13,14,16,17] and ultimately decrease hepatic ALAS1 activity. However, the cimetidine dose (200 mg/kg) used to demonstrate inhibition of ALAS and HO activities in rats markedly exceeds recommended daily dose amounts in humans [31,32]. Another limitation of these studies was that the effectiveness of cimetidine in decreasing ALA and PBG that accumulate during an acute attack was not evaluated, presumably due to the lack of a genetic mouse model for AIP at the time. 

Here, we investigated whether clinically relevant doses (15, 30, and 120 mg/kg/day) of cimetidine inhibit the hepatic activity of ALAS and HO in vivo, first in wildtype mice and then in a well-established genetic mouse model for AIP [26,33,34,35]. When the AIP mice are administered the prototypic porphyrinogenic drug phenobarbital, their hepatic ALAS1 expression is induced, and massive amounts of plasma and urinary ALA and PBG accumulate, thereby providing a biochemically relevant model of an acute porphyric attack in humans. We report that cimetidine does not effectively inhibit endogenous or induced hepatic ALAS1 activity, nor does it inhibit hepatic HO activity at the tested doses. Moreover, cimetidine does not effectively decrease plasma ALA and PBG concentrations that are elevated during an induced attack. 

Additionally, we explored whether cimetidine modulates ALAS2 activity in vitro and then in murine bone marrow cells, as cimetidine also has been proposed to be an effective treatment for erythropoietic protoporphyria (EPP) [36,37,38]. In EPP, the primary phenotype is skin photosensitivity due to decreased ferrochelatase activity that leads to accumulation of the photoreactive porphyrin protoporphyrin IX (PPIX) in bone marrow erythroid cells, where heme synthesis is mainly driven by the erythroid-specific ALAS2 isozyme (Supplemental Figure S1A). A notable improvement in cutaneous photosensitivity was reported in these studies that included 4 pediatric and 15 adult EPP patients administered cimetidine daily (30–40 mg/kg/day in children; 800–1600 mg/day in adults) [36,37,38]. While it has been speculated that cimetidine leads to clinical benefits by inhibiting bone marrow ALAS2 activity, studies conducted to date have not investigated this.

## 2. Materials and Methods

### 2.1. Animal Studies

The previously established T1/T2 AIP mice [26,33,34,35] were bred and maintained in a barrier facility at the Icahn School of Medicine at Mount Sinai (ISMMS). All animal procedures were reviewed and approved by the ISMMS Institutional Animal Care and Use Committee (IACUC). Male mice aged 6 to 12 weeks old were used for these studies, as it was previously shown that hepatic *Alas1* expression is induced to higher levels in male mice compared to females following phenobarbital administration [34]. Biochemical induction of acute attacks was performed as previously described [24,26], that is, by co-administering 3,5-diethoxycarbonyl-1,4-dihydrocollidine (DDC; 20 mg/kg/day × 4 days, oral gavage) and phenobarbital (PB; 90, 100, 110, and 90 mg/kg/day × 4 days, intraperitoneal injection). Cimetidine was administered via intraperitoneal injection at the doses indicated. Hemin (Panhematin^®^, Recordati Rare Diseases Inc., Bridgewater, NJ, USA) was prepared freshly prior to each use by dissolving it in 25% human albumin solution in amber tubes and administered via tail vein injections. Givosiran (Givlaari^®^, Alnylam Pharmaceuticals, Cambridge, MA, USA) was reconstituted according to the manufacturer’s instructions and administered as a single subcutaneous injection. Blood samples were collected via the facial vein technique using a 21G needle and centrifuged at 1000× *g* for 10 min to isolate plasma. Mice were deeply anesthetized with ketamine/xylazine and euthanized at the indicated times via cardiac perfusion with phosphate-buffered saline (PBS). Well-perfused livers and bone marrow cells were harvested, and the latter were treated with red blood cell lysis solution (Qiagen, Germantown, MD, USA) and then snap-frozen in liquid nitrogen and stored in the dark at −80 °C until use. 

### 2.2. Quantitation of Plasma ALA and PBG 

Plasma ALA and PBG concentrations were determined as previously described [26,39]. In brief, ALA and PBG were extracted and purified from plasma samples using a solid phase extraction column followed by butylation with 3 N hydrochloric acid in butanol. ALA and PBG esters were then separated using a reverse phase C8 column using a gradient program and analyzed via Agilent 6460 tandem mass spectrometer (Agilent Technologies, Santa Clara, CA, USA). Plasma ALA and PBG concentrations were expressed as μmoles/L.

### 2.3. RNA Extraction and Quantitative PCR for mRNA Quantification

RNA isolation, reverse transcription, and quantitative PCR were performed as previously described [26]. PCR was performed using predesigned Taqman assays for *Alas1* (Thermo Fisher Scientific, Waltham, MA, USA; Assay ID: Mm01235914_m1), *Alas2* (Mm00802083_m1), *Hmox1* (Mm00516005_m1), and *β-actin* (Mm02619580_g1), and transcript levels were quantitated with an ABI Prism 7900 sequence detection system. Relative mRNA levels were calculated via the comparative C_t_ method using *β-actin* as an internal control. Experiments were performed in triplicate.

### 2.4. ALAS Enzyme Activity Assay

ALAS activities were determined in murine livers, bone marrow cells, or purified recombinant human ALAS2 enzyme as previously described [40], with the following modifications. Cell pellets were sonicated in 4 volumes of 50 mM of potassium phosphate pH 7.4 (KPi), while hepatic tissue was homogenized to 20% *w*/*v* in KPi using a Potter-Elvehgem glass-teflon homogenizer. Protein concentrations were adjusted to 10 mg/mL with KPi. Samples (25 µL) were mixed with assay buffer in KPi (25 µL), reaching a final concentration of 50 mM of glycine, 100 µM of succinyl-CoA, 80 µM of pyridoxal phosphate, and 50 µM of succinylacetone. The mixtures were incubated at 37 °C for 30 min and diluted with 9 volumes of ice-cold water. A total of 50 μL of each diluted sample was mixed with 150 μL of derivatizing agent (a clear solution of water, 37% formaldehyde, ethanol, and acetone in a ratio of 107:5:15:23 by volume, respectively) and then incubated at 100 °C for 5 min. Samples were then centrifuged at 16,000× *g* for 10 min at 4 °C, and supernatants were collected and examined using Ultra Performance Liquid Chromatography (UPLC). All reactions were performed under conditions of linearity.

### 2.5. Heme Oxygenase (HO) Activity Assay 

HO activity was determined by quantitating the amount of bilirubin formed using previously described methods [41]. As shown in Supplemental Figure S1B, HO converts heme into biliverdin, which is then rapidly metabolized to bilirubin through the activity of biliverdin reductase. Briefly, mouse livers were homogenized in HO activity buffer [2 mM of MgCl_2_ and 100 mM of phosphate buffer (pH 7.4) with a protease inhibitor cocktail (Thermo Fisher Scientific)] following addition of 250 mM of sucrose and then centrifuged at 12,000× *g* for 15 min at 4 °C. The resulting supernatant was centrifuged at 105,000× *g* for 1 h at 4 °C, and the pellet (i.e., microsomal fraction) was isolated and resuspended in HO activity buffer. Bone marrow cells were homogenized in HO activity buffer and centrifuged at 18,800× *g* for 15 min at 4 °C, and the supernatant was collected for HO activity quantitation. Protein concentrations were determined using the Bio-Rad Protein Assay kit (Hercules, CA, USA).

To obtain a liver cytosol fraction, which served as the source of biliverdin reductase in the assay, livers were isolated from wildtype mice that were fasted overnight, homogenized in HO activity buffer, and then centrifuged at 105,000× *g* for 1 h at 4 °C. Assay-positive controls were obtained by intraperitoneally injecting wildtype mice with 2 mg/kg of lipopolysaccharide (LPS; Sigma-Aldrich, St Louis, MO, USA), which induces hepatic HO activity [42], and isolating liver microsomes as described above.

Reaction mixtures consisted of liver microsome or bone marrow cell homogenate (0.6 mg), 2 mg of liver cytosol, 20 mM of hemin, 0.8 mM of NADPH, 2 mM of glucose 6-phosphate, and 0.0016 U/μL of glucose-6-phosphate dehydrogenase. Reaction mixtures were incubated for 1 h at 37 °C in the dark, and reactions were terminated by placing the tubes on ice for 10 min. Absorbances at 464 nm were determined using a Synergy H1 plate reader (Biotek; Winooski, VT, USA), and bilirubin concentrations were calculated by subtracting the measured absorbance of an NADPH-free control reaction mixture (i.e., background absorbance) from each sample absorbance value and then applying a bilirubin standard curve. All reactions were performed under conditions of linearity. HO activity was represented as nanomoles of bilirubin formed per milligram of protein per hour.

### 2.6. Prokaryotic Expression and Purification of Human ALAS2

A cDNA encoding human ALAS2 without the mitochondrial targeting sequence was PCR-amplified to include an Nde I and BamHI site at the 5′- and 3′-ends, respectively. The amplicon was cloned into the pET-16b vector (Novagen, Madison, WI, USA) such that it would be in-frame with the 10× histidine tag and expressed in BL21-pLysS cells using standard growth and induction protocols [43]. The expressed protein was affinity-purified using metal chelate affinity chromatography on Ni2+-NTA resin (Thermo Fisher Scientific) and dialyzed in 50 mM of Tris at pH 6.8 plus 10% glycerol [44]. A set amount of purified ALAS2 protein (0.025 µg) was mixed with different concentrations of cimetidine, and ALAS activities were determined using methods described above.

### 2.7. Statistical Analyses

One-way analyses of variance (ANOVA) tests were performed by comparing enzymatic activities or relative gene expression levels between cimetidine-treated and control groups. Post-hoc analyses were performed using Turkey’s multiple comparison test. For ALA and PBG analyses, one-way student’s *t*-test was used. Differences between groups were considered statistically significant at a *p* value of <0.05. All statistical analyses were performed using Graphpad Prism 9.0 (Graphpad software Inc., La Jolla, CA, USA)

## 3. Results

### 3.1. Cimetidine Administration Does Not Lead to Statistically Significant Decreases in Endogenous ALAS or HO Activity in Murine Livers

**Figure 1:**
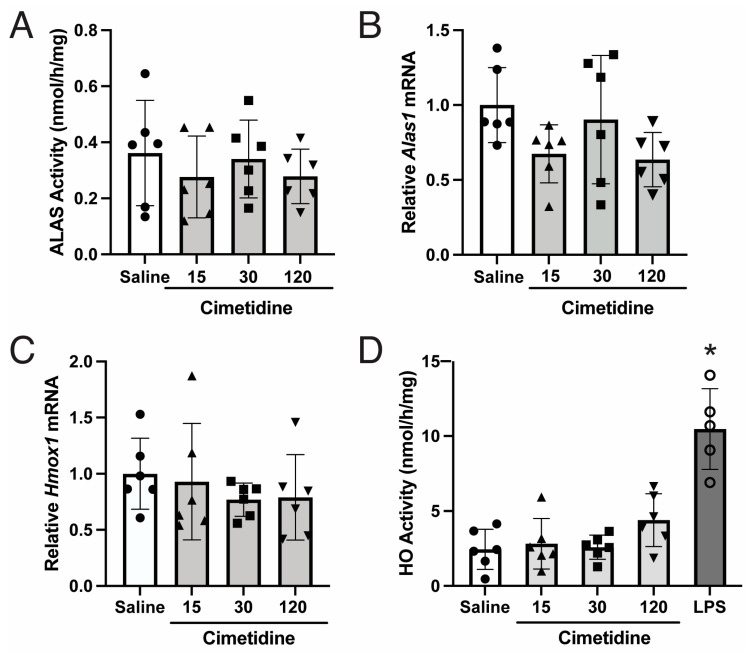
Cimetidine does not inhibit ALAS or HO activity, nor does it alter *Alas1* or *Hmox1* mRNA expression in wildtype mouse livers. Wildtype male mice were treated with four daily intraperitoneal injections of cimetidine [15 (solid up-pointing triangles), 30 (solid squares), or 120 mg/kg (solid down-pointing triangles)] or saline (solid circles) and euthanized ~2 h after the last dose. Well-perfused livers were isolated for quantitation of (**A**) ALAS activity (*p* = 0.666, one-way ANOVA) and relative mRNA expression levels of (**B**) *Alas1* (*p* = 0.102, one-way ANOVA) and (**C**) *Hmox1* (*p* = 0.652, one-way ANOVA). (**D**) Hepatic microsomal HO activities, with data from LPS-induced murine liver microsomes (open circles) included as a positive control, are shown. * *p* < 0.0001 determined via one-way ANOVA with post-hoc analyses indicating significant differences only between LPS control vs. all other groups. Data presented are means ± SDs (*n* = 6 per treatment group).

Administration of daily intraperitoneal doses of cimetidine (15, 30, or 120 mg/kg/day) for 4 days to C57BL/6J wildtype male mice led to mean hepatic ALAS activities of 0.38, 0.45, and 0.36 nmol/h/mg, respectively (Figure 1A). While these levels were slightly lower compared to the mean activity in the saline-treated controls (0.49 nmol/h/mg), the differences were not statistically significant (Figure 1A).

If cimetidine effectively inhibits hepatic HO activity, it is anticipated that heme catabolism would decrease and hepatic heme pools would increase, thereby leading to decreased hepatic *Alas1* mRNA and/or decreased import of the pre-protein into the mitochondria and decreased production of ALA. Simultaneously, the increased heme pool may induce the transcription of the inducible form of HO, *Hmox1* [45]. Following four days of cimetidine administration at doses of 15, 30, or 120 mg/kg/day, *Alas1* mRNA levels decreased by ~35%, increased by ~11%, and decreased by ~44%, respectively, compared to the saline-treated control males (Figure 1B). However, none of these changes were statistically significant (Figure 1B). *Hmox1* mRNA levels were also largely unchanged (Figure 1C).

Consistent with these findings, cimetidine treatment did not lead to statistically significant changes in hepatic microsomal HO activities compared to the saline-treated mice (Figure 1D). In fact, compared to the saline controls, which had a mean activity of 2.45 nmol/h/mg, the mice treated with 15, 30, or 120 mg/kg of cimetidine had slightly increased mean activities of 2.81, 2.59, and 4.39 nmol/h/mg, respectively (Figure 1D). 

### 3.2. Cimetidine Does Not Decrease Hepatic ALAS Activity That Is Markedly Elevated during a Biochemical Acute Attack in AIP Mice

**Figure 2:**
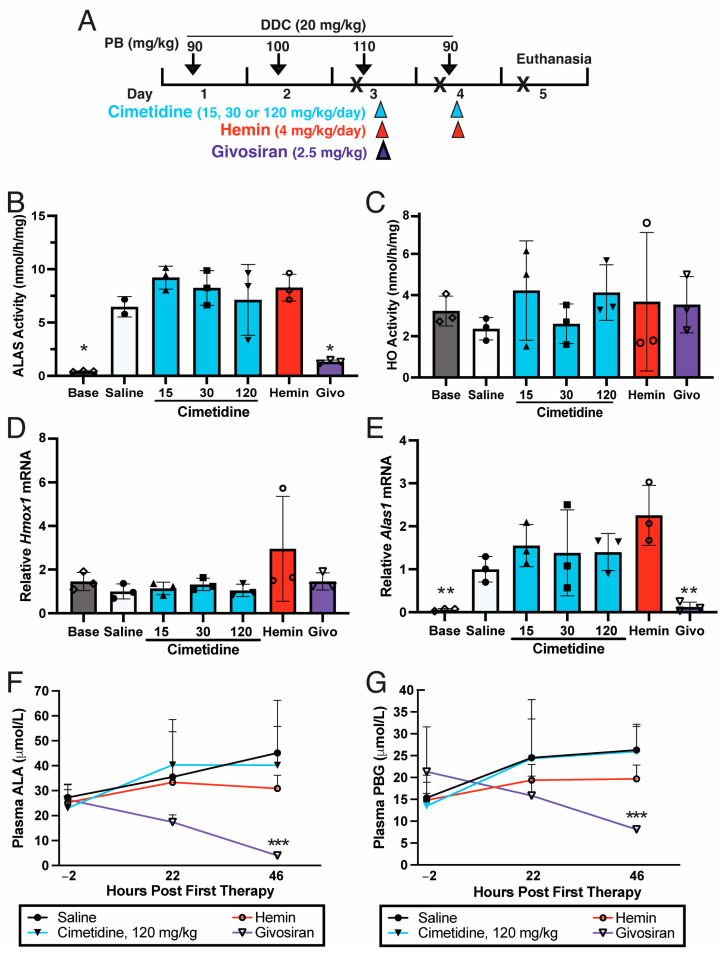
Cimetidine does not effectively decrease hepatic activities of ALAS or HO or plasma ALA and PBG that are induced during a biochemical attack in AIP mice. (**A**) Schematic of experimental setup. Biochemical acute attacks were induced in T1/T2 AIP male mice via co-administration of phenobarbital (PB, 90, 100, 110, and 90 mg/kg/day, i.p.) and 3,5-diethoxycarbonyl-1,4-dihydrocollidine (DDC, 20 mg/kg, gavage) on days 1 to 4. On days 3 and 4, mice were treated with cimetidine [15 (solid up-pointing triangles), 30 (solid squares), or 120 mg/kg (solid down-pointing triangles, i.p.)], hemin (4 mg/kg, i.v. open circles), or saline (solid circles), while another subset of mice was treated with a single dose of givosiran (2.5 mg/kg, s.c.; open down-pointing triangles) on day 3. Mice were euthanized on day 5 for quantitation of hepatic activities of (**B**) ALAS (* *p* < 0.0001 via one-way ANOVA, with post-hoc analyses showing significance only in comparisons that include givosiran or baseline groups) and (**C**) HO (*p* = 0.820, one-way ANOVA). Relative mRNA levels of (**D**) *Hmox1* (*p* = 0.253, one-way ANOVA) and (**E**) *Alas1* (** *p* = 0.0007 via one-way ANOVA, with post-hoc analyses showing significance only in comparisons that include givosiran or baseline groups) were also determined. Endogenous, baseline (Base; open diamonds) levels are shown for reference. Time course of (**F**) plasma ALA and (**G**) PBG in PB/DDC-induce AIP mice was monitored, with 0 h being time of therapeutic intervention on day 3. *** *p* < 0.05 for saline vs. givosiran, one-tailed Student’s *t* test. In panels (**B**) through (**G**), data shown are means ± SDs (*n* = 3).

Subsequent efforts assessed whether cimetidine represses induced hepatic ALAS activity and/or HO activity and ameliorates the elevation in ALA and PBG that is observed during an acute attack. As depicted in Figure 2A, T1/T2 AIP mice [33,34] were co-administered PB and DDC for four consecutive days to induce an acute attack with markedly elevated hepatic ALAS1 activity as well as plasma ALA and PBG levels [24,26]. The control T1/T2 mice induced with PB/DDC and then administered saline had mean hepatic ALAS activity of 6.47 nmol/h/mg, corresponding to an ~13.7-fold elevation compared to the baseline mean endogenous levels (Figure 2B). Administration of two daily doses of cimetidine at 15, 30, or 120 mg/kg/day to the PB/DDC-induced AIP mice resulted in mean hepatic ALAS activities of 9.20, 8.24, and 7.11 nmol/h/mg, respectively, which represent ~42%, 27%, and 10% increases compared to the saline controls (Figure 2B). The mean hepatic ALAS activity in the mice treated with two daily hemin infusions (4 mg/kg) was 8.27 nmol/h/mg, or ~28% higher than that of the saline controls. The mice treated with a single subcutaneous dose of givosiran had an average ALAS activity of 1.32 nmol/h/mg, or ~80% lower than that of the saline controls (Figure 2B). 

### 3.3. Cimetidine Does Not Inhibit HO Activity in PB/DDC-Induced AIP Mouse Livers

The PB/DDC-induced T1/T2 control mice treated with saline had mean hepatic microsomal HO activity of 2.37 nmol/h/mg, which was ~27% lower than the mean baseline levels of 3.23 nmol/h/mg (Figure 2C). *Hmox1* mRNA was also decreased by ~40% in the saline controls relative to the baseline expression levels (Figure 2D). These changes likely reflect the liver decreasing heme degradation in response to the increased heme demand brought on by the porphyrinogenic drugs PB and DDC. Administration of two daily intraperitoneal doses of cimetidine (15, 30, or 120 mg/kg/day) to the PB/DDC-induced AIP mice resulted in mean hepatic microsomal HO activities of 4.22, 2.61, and 4.12 nmol/h/mg, respectively, none of which were significantly different from those of the saline-treated controls (Figure 2C). The mean hepatic microsomal HO activities in the mice treated with two daily hemin infusions or a single subcutaneous dose of givosiran were slightly increased relative to those of the saline controls (Figure 2C). 

In line with these findings, cimetidine treatment did not decrease hepatic *Alas1* mRNA expression that was elevated by ~17-fold over baseline levels in the PB/DDC-induced mice. In fact, the hepatic *Alas1* mRNA levels were 1.6-, 1.4-, and 1.4-fold higher in the mice treated with 15, 30, or 120 mg/kg of cimetidine, respectively, compared to the saline-controls (Figure 2E). The mice treated with two hemin infusions unexpectedly had mean hepatic *Alas1* mRNA levels that were ~2.3-fold higher than those of the saline controls, whereas the mice treated with a single dose of givosiran had a mean *Alas1* level that was ~88% lower relative to the saline controls (Figure 2E). Hepatic *Hmox1* mRNA was largely unaltered by cimetidine or givosiran treatment (Figure 2D). On the contrary, two hemin infusions induced mean hepatic *Hmox1* levels to a nearly 3-fold greater extent compared to the saline controls (Figure 2D), although these differences were statistically non-significant. 

Thus, none of the tested doses of cimetidine reduced hepatic ALAS activity or HO activity in this model of acute attack. The finding that hepatic *Alas1* mRNA was essentially unaltered following cimetidine administration further supports that the drug did not meaningfully increase hepatic heme pools via the inhibition of HO activity.

### 3.4. Cimetidine Does Not Decrease Plasma ALA and PBG Levels That Are Markedly Elevated during a Biochemical Acute Attack in AIP Mice

The PB/DDC challenge markedly increased mean plasma ALA and PBG concentrations, which were 23–27 and 13–21 μmol/L, respectively, when quantitated 2 h prior to therapeutic treatment (‘pre-treatment’ levels) with cimetidine, hemin, or givosiran (Figure 2F,G). These levels represented increases of ~19–23- and 16–26-fold, respectively, compared to the mean baseline ALA and PBG levels of 1.2 and 0.8 μmol/L in uninduced AIP mice. The PB/DDC-induced control AIP mice treated with two saline injections had a mean plasma ALA concentration of 45.1 μmol/L at 46 h following the first saline injection compared to a pre-treatment level of 27.2 μmol/L, representing an increase of ~66% (Figure 2F). Two daily cimetidine doses of 120 mg/kg, the dose that achieved the lowest hepatic ALAS activity and *Alas1* mRNA levels (Figure 2B,E), led to a mean plasma ALA concentration of 40.2 μmol/L at 46 h following the first dose, corresponding to a ~74% increase relative to the pre-treatment levels of 23.1 μmol/L (*p* = 0.382 for 46 h saline vs. cimetidine, one-tailed Student’s *t* test). On the contrary, the PB/DDC-induced AIP mice that received two daily infusions of hemin had pretreatment and post-treatment levels of 25.4 and 30.9 μmol/L, respectively (Figure 2F), representing a considerably smaller 22% increase (*p* = 0.161 for 46 h saline vs. hemin, one-tailed Student’s *t* test). Notably, mice that were treated with a single subcutaneous injection of givosiran markedly and rapidly decreased their plasma ALA, with pre- and post-treatment concentrations of 26.4 and 4.0 μmol/L, corresponding to an 85% decrease (*p* = 0.011 for 46 h saline vs. givosiran, one-tailed Student’s *t* test). 

Similarly, the mean plasma PBG concentrations in the PB/DDC-induced saline controls increased by ~72%, from 15.3 to 26.3 μmol/L, when comparing pre-treatment and 46 h post-treatment levels, respectively (Figure 2G). In the cimetidine (120 mg/kg) treatment group, the mean plasma PBG concentrations at these two time points were 13.5 and 26.0 μmol/L, respectively, representing an increase of ~93% (*p* = 0.483 for 46 h saline vs. cimetidine, Student’s *t* test). The mice treated with two daily doses of hemin had a comparatively modest 33% increase in mean plasma PBG, with pretreatment and 46 h post-treatment levels of 14.8 and 19.7 μmol, respectively (*p* = 0.071 for 46 h saline vs. hemin, one-tailed Student’s *t* test). Following a single administration of givosiran, mean plasma PBG concentrations dramatically decreased from 21.3 to 8.1 μmol/L at pre- and post 46 h time points, respectively (Figure 2G) (*p* = 0.002 for 46 h saline vs. givosiran, one-tailed Student’s *t* test).

Thus, two daily injections of cimetidine did not decrease plasma ALA and PBG concentrations that were markedly elevated during an induced acute attack in the AIP mice, whereas hemin modestly decreased and givosiran markedly and rapidly decreased plasma ALA and PBG concentrations.

### 3.5. Cimetidine Does Not Alter Activity or Expression of ALAS or HO in Wildtype Murine Bone Marrow Cells

Given recent reports that cimetidine improved cutaneous photosensitivity in patients with EPP, presumably by inhibiting ALAS activity in erythroid cells [36,37,38], we investigated whether cimetidine inhibits ALAS2 activity, first in vitro and then in bone marrow cells isolated from cimetidine-treated mice. Human ALAS2 enzyme (NP_001033057.3) was prokaryotically expressed and purified, and its activity was measured in the presence of increasing concentrations (0 to 240 μmol/L) of cimetidine (Table 1). In the absence of pyridoxal-5′-phosphate (PLP), an obligatory co-factor of ALAS, the mean ALAS activity was 94 ± 6 pmol/h, and activity was essentially unchanged by the addition of cimetidine up to concentrations of 20 μmol/L. The addition of PLP (20 or 80 μmol/L) increased the overall mean ALAS activities to 170 ± 12 pmol/h and 163 ± 8 pmol/h, respectively, but increasing concentrations of cimetidine only led to decreases in ALAS activity up to 10%, which were not statistically significant (Table 1). These results strongly suggest that cimetidine does not directly inhibit the enzymatic activity of ALAS2 in vitro.

**Figure 3:**
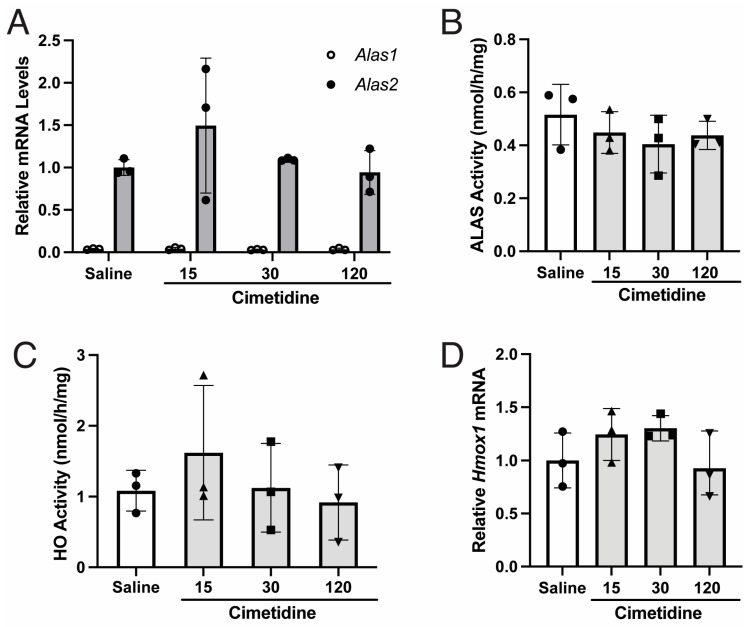
Cimetidine does not effectively decrease ALAS activity or modulate mRNA levels of *Alas1*, *Alas2*, or *Hmox1* in murine bone marrow cells. Wildtype male mice were treated with four daily intraperitoneal injections of cimetidine [15 (solid up-pointing triangle), 30 (solid square) or 120 mg/kg/day (solid down-pointing triangle) in panels (**B**) through (**D**)] and euthanized ~2 h after the last dose. Bone marrow cells were isolated and quantitated for (**A**) *Alas1* and *Alas2* mRNA; (**B**) ALAS activity (*p* = 0.542, one-way ANOVA) (**C**) HO activity (*p* = 0.597, one-way ANOVA); and (**D**) *Hmox1* mRNA (*p* = 0.236, one-way ANOVA). Data shown are means ± SDs (*n* = 3).

For the in vivo studies, we initially evaluated endogenous *Alas1* and *Alas2* mRNA expression in isolated bone marrow cells of wildtype mice. As shown in Figure 3A, the mean *Alas2* mRNA level was ~27-fold higher than that of *Alas1* mRNA in the saline controls, suggesting that ALAS2 is responsible for most of the ALAS activity in murine bone marrow cells. Treatment of the wildtype mice with four daily doses of 15, 30, or 120 mg/kg of cimetidine did not lead to statistically significant decreases in ALAS activity (Figure 3B). Mean HO activities in bone marrow cells were 1.62, 1.12, and 0.92 nmol/h/mg following the administration of 15, 30, or 120 mg/kg of cimetidine, respectively, which were not significantly different compared to the mean level in the saline controls, i.e., 1.08 nmol/h/mg (Figure 3C). mRNA levels of *Alas1*, *Alas2*, and *Hmox1* were also largely unchanged (Figure 3A,D). 

## 4. Discussion

While the underlying pathogenic mechanism of acute porphyric attacks is not fully understood, an increasing body of evidence indicates that the ALA and/or PBG that massively accumulate in the liver and are subsequently released into the plasma are neurotoxic and initiate sequelae that can result in life-threatening neurovisceral responses [18,19,20]. Thus, efficacious therapies for acute attacks need to effectively decrease hepatic production of ALA and PBG, as do hemin and givosiran (Figure 2F,G).

Here, we evaluated the therapeutic effectiveness of cimetidine in treating acute attacks. Initial efforts assessed whether administration of clinically relevant doses of cimetidine into wildtype mice decreased hepatic ALAS and HO activities, as has been previously shown by Marcus et al. in rats that were uninduced and in those that were induced with the porphyrinogenic drug allylisopropylacetamide (AIA) [31,32]. Specifically, they demonstrated a 40–45% reduction in hepatic ALAS activity and a 25–50% reduction in HO activity following a single dose of cimetidine [31,32]. Interestingly, this inhibitory effect of cimetidine on ALAS activity (presumably using recombinant ALAS1, although not specified) was not detected when tested in vitro [31]. In the present study, although cimetidine showed a tendency to decrease hepatic ALAS activity at certain doses (15 or 120 mg/kg) in wildtype mice (Figure 1A), the reductions were not statistically significant, indicating that the ability of cimetidine to decrease ALA synthesis in liver is limited, if existent. HO activity was unchanged or even slightly increased (Figure 1D). A likely reason why our results differed considerably from those of Marcus et al. is that the cimetidine dose administered by Marcus was 200 mg/kg, notably higher than doses we tested (15, 30 or 120 mg/kg). Indeed, Marcus et al. showed that rats administered 50 mg/kg or 100 mg/kg of cimetidine only decreased hepatic ALAS activity by <20% and ~30%, respectively [31]. For reference, the recommended doses of cimetidine are 800–1600 mg/day in adults, corresponding to 13–27 mg/kg/day in a 60 kg adult. Another possible reason for the discrepancy in the results between the two studies is the timing of the euthanization of the animals. Marcus et al. showed that hepatic ALAS is maximally suppressed at 1 h post-cimetidine administration and that by 90 min post-administration, it returns to baseline levels [31]. Hepatic HO activity was still suppressed by ~40% at 90 min post-administration, but further monitoring was not reported [31]. We euthanized the mice ~2 h after the last dose; therefore, it is possible that we missed these transient changes in enzymatic activity. 

Subsequently, we evaluated the therapeutic effectiveness of cimetidine using a well-established genetic mouse model for AIP, focusing primarily on its impact on hepatic ALAS activity and its ability to reduce plasma ALA and PBG concentrations. Administration of two daily doses of cimetidine into AIP mice that were induced with PB and DDC did not result in decreased hepatic ALAS activity or plasma ALA and PBG (Figure 2B,F,G). These findings contrasted to those of currently approved treatments for acute porphyric attacks. In particular, a single dose of givosiran markedly decreased hepatic *Alas1* mRNA and ALAS activity (Figure 2B,E) and significantly reduced plasma ALA and PBG within 48 h of administration (Figure 2F,G). Two daily doses of hemin also decreased plasma ALA and PBG (Figure 2F,G), although hepatic *Alas1* mRNA and ALAS activity were unexpectedly increased compared to those of saline-treated controls (Figure 2B,E). A plausible explanation is that the exogenous heme provided by hematin initially repressed *Alas1* mRNA and reduced ALA and PBG production (Figure 2F,G), but because it also induced *Hmox1* (Figure 2D) and accelerated heme degradation, there may have been rebound induction of *Alas1* at the time of sacrifice. Taken together, these findings establish that cimetidine is not an effective treatment for acute porphyric attacks.

Notably, cimetidine has also been proposed to be an effective treatment for the hepatocutaneous porphyria, porphyria cutanea tarda (PCT), based on two case reports, each with a single patient [46,47]. However, it should be noted that in PCT, it is unclear whether hepatic ALAS1 expression is induced during active disease or whether decreasing hepatic ALAS and/or HO activity would have any therapeutic benefit. Either way, our studies indicate that cimetidine does not effectively decrease hepatic ALAS or HO activity (Figure 1A,D). 

The cutaneous symptoms of EPP are believed to occur when the PPIX that is overproduced in the erythrocytes is transferred to the plasma and is taken up by endothelial cells of the superficial capillaries, where it is exposed to photons and activated. This triggers reactive oxygen species production and complement activation, both of which lead to inflammatory changes [48,49]. It has been reported that a daily oral regimen of cimetidine markedly improved cutaneous photosensitivity in four pediatric patients with EPP [37,38]. However, the EPP-causing *FECH* mutations were documented in only one of these patients [37], and PPIX was measured before and after cimetidine administration in another single patient [38]. While this patient reportedly decreased ‘erythrocyte protoporphyrin’ levels by 37% one year after initiating daily oral cimetidine [38], it was not specified whether total, metal-free, or zinc-PPIX were quantitated. This is of importance, as the diagnosis of EPP is established by demonstrating increased total PPIX, consisting predominantly of metal-free PPIX. 

A subsequent study by Heerfordt et al. reported that of 15 adult EPP patients who were administered oral cimetidine for 4 months or longer, 10 patients (67%) experienced delays in developing cutaneous symptoms following sun exposure, and 5 patients (33%) were able to tolerate longer sun exposure [36]. However, median metal-free PPIX concentrations in erythrocytes only decreased by 20% at 4 months after the initiation of treatment, and it is unclear whether this reduction is clinically meaningful. Of note, the self-reported improvement in symptoms did not necessarily correlate with levels of free-PPIX. For instance, patients #8 and #7, who had the most and third-most decreased free-PPIX levels (53% and 43% reduction, respectively) amongst all patients, reported no clear improvement in symptoms. On the contrary, patient #5, who only had a 6% reduction in free-PPIX at both 2 and 4 months, and patient #13, who actually had 18–34% increased free-PPIX, reported improved symptoms. These observations raise the possibility that cimetidine may improve cutaneous photosensitivity through a mechanism(s) that is unrelated to the heme biosynthesis/degradation pathway. Since cimetidine has been shown to exert anti-oxidant and anti-inflammatory effects [50], it is possible that the cutaneous symptoms are alleviated via these mechanisms in EPP patients. An ongoing randomized Phase 2 clinical trial (NCT05020184) is assessing the effectiveness of oral cimetidine in EPP. 

Our findings show that cimetidine does not inhibit ALAS2 activity in vitro, and while there was a tendency for decreased ALAS activity in cimetidine-treated murine bone marrow cells, the changes were non-significant (Table 1; Figure 3B). Thus, if cimetidine is effective in reducing cutaneous photosensitivity and/or PPIX concentrations in EPP patients, its mechanism of action likely is not via ALAS inhibition. Unlike ALAS1, erythroid ALAS*2* expression is not believed to be under negative feedback inhibition of heme but is rather regulated by erythroid-specific transcriptional factors (e.g., GATA1) and iron availability [51,52,53]. Since heme synthesis is primarily driven by ALAS2 in bone marrow erythroid precursors, downregulation of ALAS1 via inhibition of HO in bone marrow would likely have minimal therapeutic benefit for EPP. Regardless, we show that HO activity, as well as *Alas1*, *Alas2*, and *Hmox1* mRNA in murine bone marrow cells were all largely unaltered by cimetidine (Figure 3A,C,D).

The well-established EPP mouse model (BALB/C-Fechm1Pas mice) with ~6% of normal FECH activity accumulate massive amounts of PPIX and exhibit marked photosensitivity following exposure to a mercury vapor lamp, which mimics sunlight exposure [54]. In the future, it would be of interest to treat these EPP mice with cimetidine and to evaluate its impact on the cutaneous photosensitivity and PPIX levels, particularly if ongoing clinical trials show that cimetidine is indeed efficacious in improving cutaneous photosensitivity in human EPP patients.

Thus, our studies indicate that clinically relevant doses of cimetidine do not effectively inhibit ALAS activity in liver or bone marrow, the former contributed by ALAS1 and the latter predominantly by ALAS2. Not only do ALAS1 and ALAS2 catalyze the same reaction but they also have highly similar gene structures and are believed to have emerged from gene duplication. The catalytic cores of the two human enzymes are 75% identical in amino acid sequence [55] and are the same length. In line with our findings, the structure of cimetidine has limited similarity to the ALAS substrates, glycine and succinyl-CoA, as well as to ALA and other heme intermediates (Supplemental Figure S2). 

In summary, our data indicate that cimetidine is not an effective treatment for acute porphyric attacks and that it should not replace approved proven therapies such as hemin and givosiran. While it remains to be determined whether cimetidine provides clinical benefit for patients with EPP, our studies strongly suggest that any therapeutic effects it may exert are not due to suppression of bone marrow ALAS activity. 

## Figures and Tables

**Table 1:** Cimetidine does not effectively inhibit ALAS2 activity in vitro. PLP, pyridoxal-5′-phosphate; ALAS, 5-aminolevulinic acid synthase.

Cimetidine Concentration (μmoles/L)	PLP Concentration (μmoles/L)	ALAS Activity (Mean ± SD, pmol/h)
0	0	94 ± 6
0.06	0	102 ± 4
0.2	0	96 ± 5
0.6	0	101 ± 2
2	0	100 ± 10
6	0	104 ± 9
20	0	95 ± 5
0	20	170 ± 12
0.2	20	176 ± 15
0.6	20	170 ± 9
2	20	166 ± 3
6	20	164 ± 16
20	20	152 ± 18
60	20	154 ± 13
0	80	163 ± 8
0.8	80	155 ± 3
2.4	80	170 ± 5
8	80	165 ± 7
24	80	161 ± 0.3
80	80	158 ± 9
240	80	157 ± 11

## Data Availability

The data that support the findings of this study are available from the corresponding author, [M.Y.], upon reasonable request.

## Supplementary Materials

The following supporting information can be downloaded at: https://www.mdpi.com/article/10.3390/biom14010027/s1, Figure S1: Mammalian Heme Biosynthetic and Degradation Pathways; Figure S2: Structure of Cimetidine.
