# Supplementary material for: Cimetidine Does Not Inhibit 5-Aminolevulinic Acid Synthase or Heme Oxygenase Activity: Implications for Treatment of Acute Intermittent Porphyria and Erythropoietic Protoporphyria
Source: Biomolecules. 2023 Dec 24;14(1):27. doi: 10.3390/biom14010027 (PMC10813085; doi:10.3390/biom14010027)
Supplement: Supplementary file 1 [file biomolecules-14-00027-s001.zip › biomolecules-2757075-supplementary.pdf]

Supplementary Files For Yasuda et al. Biomolecules 2757075

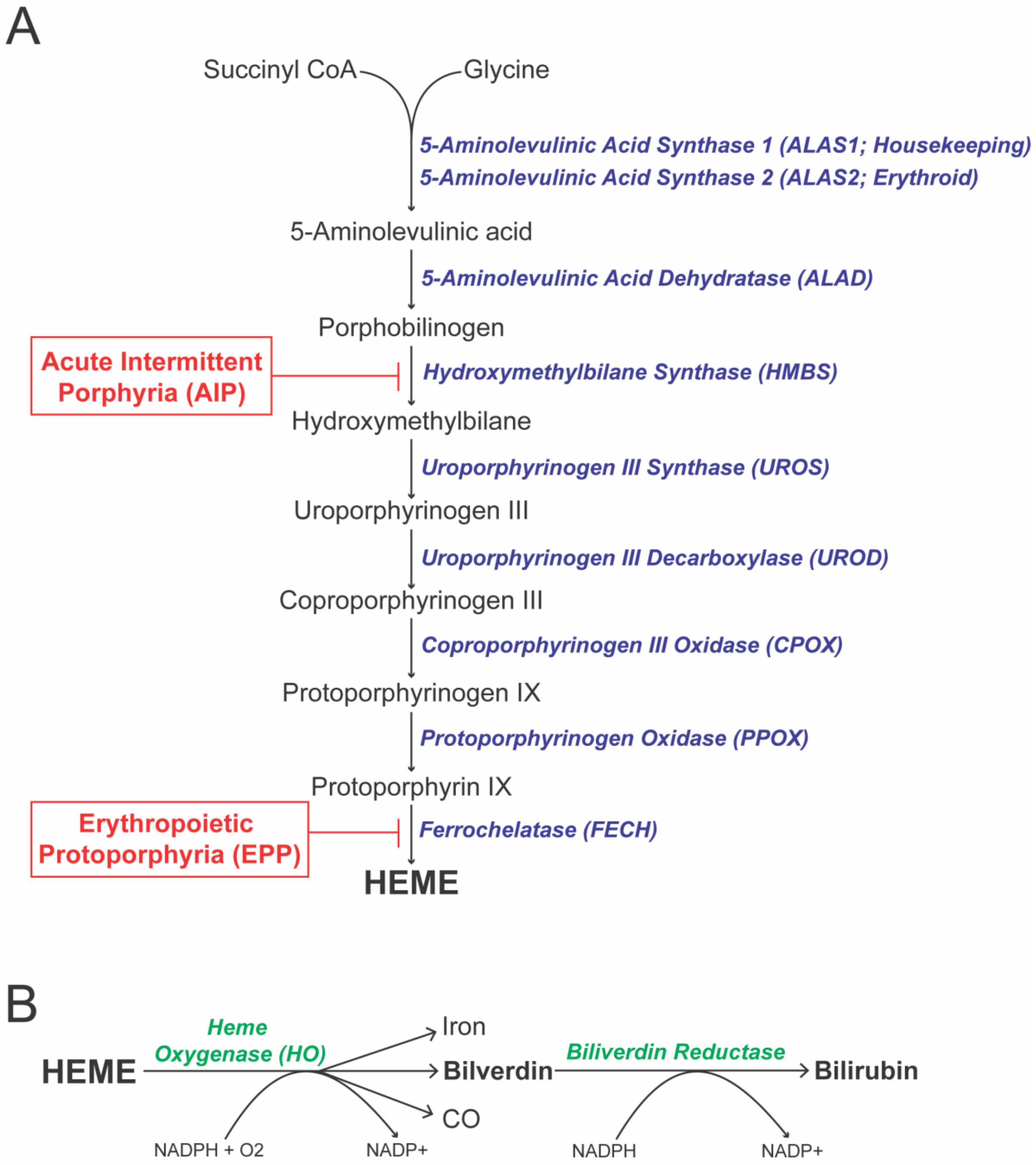

Supplemental Figure S1. Mammalian Heme Biosynthetic and Degradation Pathways. (**A**) Heme is synthesized from succinyl CoA and glycine through the action of eight enzymatic reactions, shown in blue italics. The first and rate-limiting enzyme, 5-aminolevulinic acid synthase (ALAS), has housekeeping (ALAS1) and erythroid-specific (ALAS2) isozymes. Defects in the third and last enzymes, hydroxymethylbilane synthase (HMBS) and ferrochelatase (FECH), respectively, lead to acute intermittent porphyria (AIP) and erythropoietic protoporphyria (EPP), as indicated in red. (**B**) Heme is degraded into equimolar of biliverdin, carbon monoxide (CO) and iron by the action of heme oxygenase (HO), which have inducible (HO1, encoded by *HMOX1*) and constitutive (HO2, encoded by *HMOX2*) isozymes. Biliverdin is rapidly metabolized into bilirubin by biliverdin reductase.

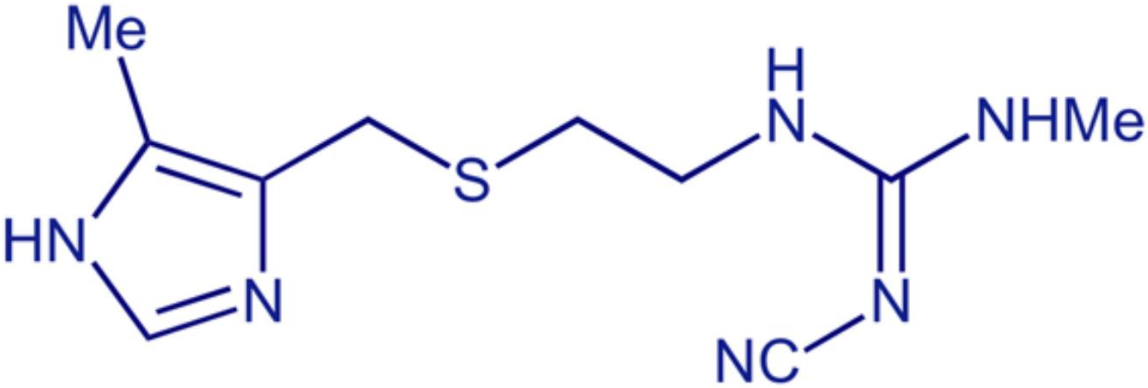

Supplemental Figure S2. Structure of Cimetidine. There is limited structural similarity to the ALAS enzyme substrates, glycine or succinyl-CoA, as is there to 5-aminolevlulinic acid (ALA) or other heme intermediates. Structure from https://www.stereoelectronics.org/webDD/DD601_cimetidine.html
